# Contraception as a risk factor for urinary tract infection in Port Harcourt, Nigeria: A case control study

**DOI:** 10.4102/phcfm.v3i1.207

**Published:** 2011-04-21

**Authors:** Paul O. Dienye, Precious K. Gbeneol

**Affiliations:** 1 Department of Family Medicine, University of Port Harcourt Teaching Hospital (UPTH), Nigeria

## Abstract

**Background:**

The concerted effort of government and donor agencies to limit fertility by the use of contraceptives has been reported in some studies to predispose to urinary tract infection (UTI). Similar studies have not been conducted in the General Outpatient Department (GOPD) of the University of Port Harcourt Teaching Hospital (UPTH).

**Objectives:**

This study was aimed at assessing the role of contraceptives in the development of UTI amongst adult females attending the GOPD of UPTH.

**Method:**

A case control study in which contraceptive users who attended the GOPD of the UPTH in four months, and an equal number of age-matched controls, were screened for UTI. The information obtained from them was entered into a specially designed pre-tested questionnaire for analysis. The results were analysed using SPSS version 14.

**Results:**

A total of 150 contraceptive users and controls were evaluated. Their age range was 18–50 years, with a mean of 27.8 ± 5.3 years. Most of the participants belonged to the lower socioeconomic classes. The combined prevalence of UTI amongst the contraceptive users and the controls was 23.7%, with the contraceptive users at 35.3% and the controls at 12.0%. The association of UTI with contraceptive use was statistically significant, with McNemar's χ^2^ = 16.28, *p* = 0.000, odds ratio (OR) = 2.9, 95% confidence interval (CI) = 1.7 – 5.3, attributable risk (AR) = 23.3, population attributable risk (PAR) = 11.7. The users of barrier contraceptives were more predisposed to UTI (OR = 17.30, 95% CI = 7.49 -39.96).

**Conclusion:**

Contraceptive use is a significant risk factor for acquiring urinary tract infection, with the barrier methods being more predisposing. Health education for the hygienic and safe use of family planning methods will prevent long-term complications.

## Introduction

One of the most serious problems that developing countries still have to solve is the rapid and uncontrolled increase in population.^1^ Although it has been estimated that the population of these countries, including Nigeria, will double in the next thirty years,^2^ there is a high incidence of unwanted pregnancies and abortion amongst sexually active Nigerian adolescents as a result of limited access to family planning services.^3^ To stem this trend, there are concerted efforts by government and donor agencies to limit fertility through the use of contraceptives.^4^

Worldwide, contraceptive use has increased substantially over the past two decades, with improvements in existing contraceptive methods and the development of several new, more effective and acceptable methods with fewer side effects.^5^ The efforts to improve contraceptive usage are commendable, but there has been increased concern about their safety.^6^ Many studies highlight the side effects and complications of different contraceptive methods.^7, 8, 9, 10^ These studies mostly looked at hormonal contraceptives in which nausea, high blood pressure, varicose veins, menstrual disorders and breast cancer were reported.^7^

Previous studies identified urinary tract infection as a complication of contraceptive use.^11, 12^ The predisposition of women to urinary tract infection (UTI), facilitated by the heavy colonisation of their lower vagina and periurethral area by uropathogenic bacteria,^13, 14^ is aggravated by contraceptive use.

Even though UTI is a cause of morbidity, mortality and great economic loss,^15, 16^ there is no known prospective study that has determined the role of contraceptives as a risk factor for urinary tract infection in the general outpatient department (GOPD) of the University of Port Harcourt Teaching Hospital (UPTH). This study is aimed at addressing this gap in knowledge.

### Research significance

Although UTI has been known as a cause of morbidity, mortality and great economic loss, there is no known prospective study to determine the predisposing role of contraceptives in the general outpatient department (GOPD) of the University of Port Harcourt Teaching Hospital. This study will therefore fill this gap in knowledge and justify the need to intensify health promotion and education on contraceptive use.

## Ethical considerations

Ethical approval was obtained from the ethics committee of the University of Port Harcourt Teaching Hospital before the commencement of this study.

## Method

### Design

This was a matched case-control study in which consenting adult females (18–50 years old) who attended the GOPD in the UPTH, Port Harcourt within the four-month study period were recruited.

### Subject selection

All consenting adult female contraceptive users within the age bracket of 18–50 years were recruited. Pregnant patients; patients with diabetes; patients experiencing vaginal discharge, dysuria, lower abdominal pains, loin pains; patients identifying with antimicrobial use during the last 14 days; patients who have participated in sexual intercourse within the last 24 hours and those hospitalised or catheterised during the four weeks before enrolment were excluded from the study.

### Sampling method

One in every two qualified patients who presented to the GOPD, University of Port Harcourt Teaching Hospital was recruited into the study. Consecutive contraceptive users were recruited from amongst this group of patients by specifically asking them questions about their utilisation of contraceptives, sexual activity and frequency of sexual activity, as well as history of pain on micturation, loin pains and lower abdominal pains. For each contraceptive user identified, an age- and marital status-matched non-contraceptive user was selected randomly.

A total of 1504 pre-tested numbered interviewer-administered questionnaires were completed for all the recruited participants by the two researchers and two research assistants who were recruited as part of the study after being taught how to fill in the questionnaires and conduct the interviews. The questionnaire asked participants about their socio-demographic characteristics such as age, occupation and educational status, sexual history (activity and frequency) and types of contraceptives used, and symptoms of UTI such as dysuria and loin pain. Socioeconomic classes were determined using the method of Oyedeji.^17^ Cases and controls were clinically examined by the researchers. To maintain anonymity, the names of the participants were not written on the questionnaire but were coded serially. The participants were assured of confidentiality and informed that the information would only be used for scientific purposes.

The participants were requested and instructed to collect about 15 mL of ‘clean catch’ mid-stream urine in sterile bottles; this sample was sent to the microbiology laboratory of the teaching hospital for microscopy, culture and sensitivity. Those with positive bacterial culture in their urine were treated on the basis of the result.

### Analysing

The data retrieved from the questionnaires were analysed using the Statistical Package for Social Sciences (SPSS) version 14 and the Microsoft Excel (MS) software program. The proportions of patients (prevalence) with urinary tract infections were calculated as a percentage. The degree of association of urinary tract infection with contraceptive use and the type of contraception were determined using McNemar's Chi-square test, odds ratio (OR) and attributable risk (AR). Tables were constructed to present the results. Statistical significance was set at the 95% confidence level (CI, confidence interval) or at a *p*-value of less than or equal to 0.05 (*p*-value ≤ 0.05).

## Results

From the 1504 women recruited during the study period, 150 (9.97%) contraceptive users were identified. The contraceptives used were barrier methods, Billing's method, implants, injectables, intrauterine contraceptive device (IUCD), oral contraceptives, bilateral tubal ligation (BTL) and the withdrawal method. The barrier contraceptives used included male condoms [47 (82.46%)], female condoms [3 (5.26%)], foaming spermicide tablets [6 (10.53%)], and the diaphragm [1 (1.75%)]. The participants’ ages ranged from 18–50 years, with a mean age of 27.8 ± 5.3 years. The majority of the participants [63 (42.0%)] belonged to the 21–30-year age group, and 117 (78%) were married (Table 1). It was found that 35.3% of the contraceptive users and 12.0% of the controls had UTI. There was a significant association of UTI with contraceptive use (McNemar's χ2 = 16.28, *p* = 0.000, OR = 2.9, 95% CI = 1.7 – 5.3, AR = 23.3, PAR = 11.7) (Table 2). The prevalence of UTI in the various socioeconomic classes was similar amongst the contraceptive users, but amongst the controls it was higher in classes 2–4. The differences in the prevalence amongst the contraceptive users and the controls were statistically significant in all the groups, apart from classes 3 and 4 (*p* < 0.05) (Table 3). The prevalence of UTI amongst the barrier contraceptive users was 71.9% and they were found to be more predisposed to UTI (OR = 17.30, 95% CI = 7.49 – 39.96). The association between the type of contraceptive and UTI was statistically significant (*p* = 0.000) (see Table 4).

**TABLE 1:** Distribution of contraceptive users and controls by age and marital status (*n* = 150).

Distribution	Participants		Controls	
	
	*n*	*%*	*n*	*%*
**Age range**				
≤ 20	32	21.3	32	21.3
21–30	63	42.0	63	42.0
31–40	45	30.0	45	30.0
41–50	10	6.7	10	6.7
**Marital status**				
Single	26	17.3	26	17.3
Married	117	78.0	117	78.0
Widows	7	4.7	7	4.7

**TABLE 2:** The prevalence of urinary tract infection amongst contraceptive users and the controls.

UTI status	Participants		Controls	
	
	*n*	*%*	*n*	*%*
Infection	53	35.3	18	12.0
No infection	97	64.7	132	82.0

**Total**	**150**	**100**	**150**	**100**

UTI, urinary tract infection. McNemar's χ^2^ = 16.28; *p* = 0.000; odds ratio = 2.9; 95% confidence interval = 1.7 – 5.3; attributable risk proportion = 23.3; population attributable risk proportion = 11.7.

**TABLE 3:** Distribution of urinary tract infection amongst the participants from different socioeconomic classes.

Socioeconomic class	Participants		UTI		Controls		UTI		*p*-value
			
	*n*	*%*	*n*	*%*	*n*	*%*	*n*	*%*	
1	10	6.7	3	30.0	6	4.0	0	0.0	< 0.01^a^
2	19	12.7	7	36.8	5	3.3	1	20.0	0.03^a^
3	36	24.0	13	36.1	11	7.3	3	27.3	0.20
4	33	22.0	9	27.3	38	25.3	8	21.1	0.37
5	52	34.7	21	40.4	90	60.0	6	6.7	< 0.01^a^

**Total**	**150**	**-**	**53**	**-**	**150**	**-**	**18**	**-**	**-**

UTI, urinary tract infection.

a χ^2^ statistically significant.

**TABLE 4:** Association of urinary tract infection with type of contraceptive.

Type	Infection		No infection		Total
	
	*n*	*%*	*n*	*%*	
Barrier contraceptives	41	71.9	16	21.8	57
Hormone contraceptive	12	12.9	81	87.1	93

**Total**	**53**	**35.3**	**97**	**64.7**	**150**

Values are given as χ^2^ = 53.8; *p* = 0.000; odds ratio = 17.30; 95% confidence interval = 7.49 – 39.96.

**TABLE 5:** Estimates of various statistical parameters.

Statistical parameters	Estimates			

	*%*	*p*-value	Ratio	Proportion
**Prevalence of UTI**				
Amongst contraceptive users	35.3	-	-	-
Amongst non-contraceptive users	12.0	-	-	-
McNemar's Chi-square	-	16.28*	-	-
Odds ratio and 95% confidence interval	-	-	2.9 (1.7-5.3)	-
ARP	-	-	-	0.66
PARP	-	-	-	0.49

UTI, urinary tract infection; ARP, attributable risk proportion; PARP, population attributable risk proportion.

* *p* = 0.000

## Discussion

This study discovered a predominance of participants within the 16–45-year age bracket and those that were married amongst the contraceptive users. This predominance can be explained by the fact that this is the reproductive age bracket, when sexual activity is a norm. The reduced number of contraceptive users in the older age group could be explained by the fact that, with aging, there is a decline in ovarian hormonal secretion during the menopausal transition, which may alter libido, sexual response and functioning,^18, 19, 20^ with concomitant loss of interest in contraception.

The widely used indicators of socioeconomic status include education, occupational status and income.^17^ The contraceptive users in the low socioeconomic classes may be more prone to UTI due to the interplay of these social indicators.^15, 21^ The expected trend of a high prevalence of UTI in the lower socioeconomic classes was not observed in our study population, and this is not in agreement with previous reports.^22, 23^ It could be explained by the fact that the social indicators did not alter our participants’ exposure to the factors responsible for UTI amongst contraceptive users. The relevance of this finding is that, in planning health promotional programmes, equal attention should be given to people in all the socioeconomic classes.

The overall prevalence of UTI (35.3%) amongst the contraceptive users was high. There was about a three-fold increased risk of the development of urinary tract infection amongst patients who were on contraceptives compared to non-users. This finding is consistent with that of other researchers.^24, 25, 26, 27^ The fact that the participants were asymptomatic makes it an unacceptable public health problem that calls for urgent intervention, in terms of health education and promotion and encouragement of the use of contraceptive methods that carry lesser risks of urinary tract infection.

The high prevalence of UTI amongst the barrier contraceptive users in this study is in agreement with earlier studies that reported high prevalence of UTI amongst patients who used both the diaphragm with spermicide and spermicide-coated condoms.^28, 29, 30, 31, 32, 33^ Exposure to spermicides alone has been reported to increase the risk of vaginal colonisation and bacteriuria with *Escherichia coli* (*E. coli*), but not to the degree seen with the use of a diaphragm and spermicide.^25^ In the population studied here, the use of diaphragm and spermicide in the form of foaming tablets was not popular, hence the low prevalence of their usage. The high prevalence of UTI amongst the barrier contraceptive users may therefore emanate from unhygienic conditions during application of the condom,^29^ which was the commonest barrier method used in this study. Secondly, unlubricated condoms may abrade the vaginal wall and make it vulnerable to infections. Thirdly, it has been suggested that the users of the barrier methods are likely to have increased vaginal fluid pH, alterations in normal vaginal flora, and increased rates of introital colonisation with *E. coli* – all associated with UTI.^28^

The part played by the hormonal contraceptives in the aetiology of UTI was to a lesser degree than that reported in a study by Ziaei and colleagues.^26^ The effects of progesterone on muscle tone, peristalsis of the ureters and also on the urinary vasculature may account for the UTI in women who use hormonal contraceptives.^25^

## Conclusion

On the basis of the findings of this study it can be concluded that contraceptive use is a significant risk factor for acquiring urinary tract infection, with the barrier methods being more predisposing. This warrants greater attention being paid to the reproductive health needs of the women, and health education for the hygienic or safe use of family planning methods. Women who use the barrier methods could be advised to consider alternative methods, such as oral contraceptives. It is important, however, that the advantages of a method in terms of UTI prevention be balanced against the loss of protection against sexually transmitted diseases conferred by barrier methods.
